# Prevalence and Risk Factors of Prolonged QTc Interval among Chinese Patients with Type 2 Diabetes

**DOI:** 10.1155/2012/234084

**Published:** 2012-12-25

**Authors:** Xiang Li, Hui Ren, Zhang-rong Xu, Yan-jun Liu, Xiao-pin Yang, Jian-qin Liu

**Affiliations:** Diabetes Center, The 306th Hospital of PLA, Beijing 100101, China

## Abstract

*Objectives*. The aim of this study was to evaluate the prevalence and the risk factors of prolonged QTc interval among Chinese patients with type 2 diabetes. *Methods*. The retrospective study included 3156 outpatients from the Diabetes Centre, the 306th Hospital of PLA, during the period from September 2003 to June 2010. QT interval was measured manually in the 12-lead conventional electrocardiogram. The QT interval corrected for heart rate (QTc) was calculated using Bazett's formula. Additional demographic and laboratory data were also collected. Potential risk factors of prolonged QTc interval were assessed using multivariable regression. *Results.* The prevalence of prolonged QTc interval among Chinese patients with type 2 diabetes was 30.1%. Height (OR 0.156, 95% CI 0.032*~*0.748), waist circumference (OR 1.025, 95% CI 1.010*~*1.040), diastolic blood pressure (OR 1.016, 95% CI 1.007*~*1.026), postprandial glucose (OR 1.040, 95% CI 1.022*~*1.059), fasting insulin (OR 1.014, 95% CI 1.003*~*1.025), and presence of microalbuminuria (OR 1.266, 95% CI 1.033*~*1.551) were significant risk factors. *Conclusions*. The prevalence of prolonged QTc interval among Chinese patients with type 2 diabetes is high. Risk factors for prolongation of QTc interval were low height, high waist circumference, increasing diastolic blood pressure levels, high postprandial glucose levels, high fasting insulin levels, and presence of microalbuminuria.

## 1. Introduction

Cardiac autonomic neuropathy (CAN) is a serious and common complication of diabetes. It is associated with a variety of adverse outcomes including cardiovascular death [1, 2]. However, it is often overlooked by the physicians due to its insidious onset and not routinely tested in most diabetic clinics. Although several noninvasive tests, such as a battery of cardiac autonomic reflex tests [3], twenty-four-hour heart rate variability (HRV), spontaneous baroreflex sensitivity, and cardiac radionuclide imaging are currently used for the diagnosis of CAN [4], these tests are laborious and time consuming. Therefore, they are not practical screening methods for the large number of patients with diabetes mellitus.

 The QT interval reflects the duration of the ventricular myocardial depolarization and repolarization. Prolongation of the corrected QT interval (QTc) has been demonstrated to be a specific indicator of CAN in most studies [5–7]. Moreover, it is predictive of all-cause and cardiovascular mortality in both healthy population [8] and patients with diabetes [9–12]. Thus, QTc prolongation could be utilized as a rapid objective method to target the people at high risk of cardiovascular events. In spite of the reported prevalence of QT prolongation as high as 26% in patients with type 2 diabetes [13], little is known about the problem in China. In this study, we retrospectively examined the patients with type 2 diabetes attending the diabetic clinic of the 306th Hospital from 2003 to 2009 to determine the prevalence and the potential risk factors for prolonged QTc interval.

## 2. Subjects and Methods

### 2.1. Subjects

A total of 3156 patients with type 2 diabetes who had standard ECGs record from September 2003 to June 2010 were recruited. Type 2 diabetes was diagnosed according to the 1999 WHO criteria [14]. Inclusion criteria required that patients with type 2 diabetes were free of clinically apparent macrovascular and heart disease, chronic renal failure, and hypoglycemia or acute illness in the previous 24 h, had normal serum creatinine and electrolyte levels, and had not been treated with medications that may affect QT interval.

### 2.2. Baseline Methods

Medical documentations of all the patients were systematically reviewed with respect to age, sex, body weight, height, waist circumference, hip circumference, smoking habits, diabetes duration, and presence of diabetic complications including retinopathy, nephropathy, and peripheral neuropathy. Baseline laboratory data including fasting and postprandial blood glucose and plasma insulin, HbA1c, creatinine, lipids (total cholesterol, high-density lipoprotein cholesterol (HDL-C), low-density lipoprotein cholesterol (LDL-C), and triglycerides), and urine albumin-to-creatinine ratio (UACR) were also collected.

Blood pressure was measured two consecutive times 1 min apart in the sitting position using an appropriate cuff size. The mean value of the two measurements was used in the statistical analysis. Hypertension was diagnosed as systolic blood pressure (SBP) ≥140 mmHg or diastolic blood pressure (DBP) ≥90 mmHg or the current use of antihypertensive drugs.

Diabetic retinopathy was evaluated by an ophthalmologist; normoalbuminuria was defined as UACR <30 mg/g, microalbuminuria as UACR between 30 and 300 mg/g, and macroalbuminuria as UACR >300 mg/g. Clinical nephropathy was diagnosed as the patient had persistent proteinuria of 500 mg/24 h, serum creatinine above 1.5 mg/dL, or UACR above 300 mg/g for at least twice. Peripheral neuropathy was diagnosed on the basis of neuropathic symptoms and signs or objectively abnormal results including insensitivity to a 10 g monofilament and abnormal vibration perception threshold based on the biothesiometer and without other significant disease.

QT intervals and the preceding RR intervals were measured on the resting ECG tracing in lead II. The QT interval was measured manually from the starting point of QRS complex to the terminal point of the downslope of the T wave. QTc was calculated according to Bazett's formula [15]: QTc = QT/(RR)^1/2^. The QTc interval >0.44 s was considered abnormally prolonged.

### 2.3. Statistical Analysis

The normality of the distribution of each continuous variable was assessed using the Kolmogorov-Smirnov test. If normality was established, continuous variables are presented as means ± SD and student's *t*-test was used to assess the differences. Data with skewed distribution were expressed as median (quartile) and Mann-Whitney *U* test was used. Categorical variables were presented as number and percentage and Chi-square test was used to test for group difference. Variables that were found to have a significant association in univariate analyses were entered in the multivariate analyses models (stepwise backward method). Entry and removal probabilities for stepwise were 0.05 and 0.1, respectively. All analyses were performed using SPSS 17.0 software (Windows version 17.0, SPSS Inc., Chicago, IL, USA). *P* values less than 0.05 were considered significant.

## 3. Results

### 3.1. Baseline Characteristics of the Study Population

The 3156 patients with type 2 diabetes included 1425 (45.2%) females and 1731 (54.8%) males. The mean age (SD) was 54.9 (10.8) years. The median duration of diabetes was 3.0 years (0.75, 8). Of the study population, 1121 (35.5%) were smokers; 2035 (38.5%) had hypertension. Retinopathy, neuropathy, and microalbuminuria were present in 422 (13.4%), 1159 (36.7%), and 718 (22.8%) patients, respectively.

### 3.2. Prevalence of QTc Interval Prolongation and Clinical Characteristics according to QTc Interval

A prolonged QTc interval was found in 30.1% (*n* = 951) of patients. Sex difference was evident, with higher prevalence in females than in males. Patients with prolonged QTc interval had more often lower height, higher waist and hip circumferences, higher systolic and diastolic blood pressure levels, longer diabetes duration, worse glycemic control (higher postprandial glucose and HbA1c levels), worse lipid profile (higher serum concentrations of total cholesterol and LDL cholesterol levels), and higher UACR. Additionally, patients with prolonged QTc interval had microalbuminuria more often than patients with normal QTc interval. No differences in prevalence of either retinopathy or neuropathy were observed between the two groups (Table 1).

### 3.3. Prolonged QTc Interval Risk Factors

In multivariate regression analysis, after controlling for age and gender, the odds of prolonged QTc interval increased significantly with a lower height, higher waist circumference, increasing diastolic blood pressure levels, higher postprandial glucose levels, higher fasting insulin levels, and presence of microalbuminuria (Table 2).

## 4. Discussions

QTc interval represents an index of myocardial refractoriness and electrical stability. Its prolongation was associated with ventricular fibrillation and cardiac sudden death [16]. Prevalence of prolonged QT interval is higher in patients with type 1 or type 2 diabetes as compared to patients without diabetes [5, 13, 17]. The prevalence of QT prolongation has been reported to be as high as 16% in type 1 [5] and 26% in type 2 diabetes [13]. Here we report that the prevalence of prolonged QTc interval was 30.1% in Chinese patients with type 2 diabetes.

Several risk factors of prolonged QTc interval among patients with diabetes have been cited in the literature including age [17, 18], gender [19], components of insulin resistance syndrome such as BMI [20, 21], hypertension [5, 17–20, 22], insulin concentration [21, 23, 24], hyperglycemia [16, 25], diabetic microvascular complications such as diabetic retinopathy [19], neuropathy [19] and microalbuminuria, and preexisting coronary heart disease [18, 20]. Although there are inconsistencies among studies regarding all the various risk factors, hypertension was identified by most studies as an independent risk factor. In our analysis, the presence of hypertension, especially increasing diastolic blood pressure, led to a significant higher rate of prolonged QTc interval, which was in accordance with previous studies.

Hypertension is frequently associated with left ventricular hypertrophy and also associated with sympathovagal imbalance characterized by vagal withdrawal and relative sympathetic dominance [26, 27]. Structural changes in the hypertrophied myocardium, altered ion channels operating during the early repolarization phase, and fibrotic changes in the myocardium due to left ventricular hypertrophy may lead to the prolongation of QTc interval [28, 29]. Studies also have shown that sympathetic activation results in QTc interval prolongation, whereas parasympathetic activation protects against prolongation of the QTc interval [30]. Thus, the sympathovagal imbalance in hypertensive patients could also contribute to prolongation of QTc interval.

Acute hyperglycemia has been shown to increase QT interval in newly diagnosed type 2 diabetic patients [31]. In this study, postprandial glucose level was also found to be an independent risk factor for prolongation of QTc interval. The mechanisms involved in the prolongation of QTc interval during hyperglycemia were increased in intracellular calcium concentration and the impairment of the sympathovagal balance. Hyperglycemia increases the free radical production and reduces the nitric oxide (NO) production [32, 33]. The NO reduction could cause the inhibition of Ca^2+^-ATPase and K^+^/Na^+^ ATPase activity, leading to an increase of cytosolic free calcium and a prolongation of myocardial repolarization [16]. Moreover, hyperglycemia has been demonstrated to produce an increase of sympathetic activity as evident by increased plasma catecholamine concentrations [31, 34, 35]. Sympathetic stimulation unopposed by vagal activity may also induce ventricular electrical instability.

A significant positive correlation between QTc and fasting insulin level was also found in our study. This finding is in keeping with the findings of Kazumi et al. [24]. In the study of Takebayashi et al. [36], insulin therapy was also demonstrated to significantly increase QTc interval. Interestingly, in the ACCORD study [37], as compared with the standard-therapy group, patients who received intensive glucose lowering therapy (a higher percentage of insulin therapy and a higher dose of insulin) had a relative increase in mortality of 22%. Thus, prolongation of QTc interval may be one of the factors contributing to the high rate of death in the intensity-therapy group.

The detailed mechanism of induction of QTc prolongation by insulin is unknown; however, several factors have been suggested. Stimulation of cellular potassium uptake is the common mechanism for both insulin-induced hyperpolarization and insulin-induced hypokalemia [38]. Hyperpolarization prolongs the repolarization phase either by increasing the temporal dispersion of action potential recovery or through early after depolarization, which leads to prolongation of QT interval. Hypokalemia could mediate adrenergic activation and sympathetic overactivity would also lead to prolongation of QTc interval. Furthermore, in nondiabetic elderly, van Noord et al. showed that the prolongation of QTc interval is due to shortening of RR interval associated with hyperinsulinemia [39].

Our data also showed a strong independent relationship between microalbuminuria and QTc interval prolongation. The relationship between QTc interval and microalbuminuria has been described both in type 1 diabetes [5, 40, 41] and type 2 diabetes [42]. However, the pathophysiological basis for the association is unclear. Although the presence of microalbuminuria was associated with QT prolongation in the study of Rutter et al., QT prolongation was not strongly linked to albumin excretion rate but more strongly to other factors such as systolic blood pressure and factor XIIa [43]. These findings support the hypothesis that microalbuminuria and QT prolongation have common determinants. Meanwhile, patients with proteinuria have a higher prevalence of coronary heart disease [44] and autonomic neuropathy [45], which, in turn, are both related to prolongation of QTc interval.

It has been shown that high BMI is associated with QTc interval prolongation [18, 46]. However, in the current study, it was not high BMI, but low height and high waist circumference were significantly associated with prolongation of QTc interval. This was in accordance with the findings of Takebayashi et al. [36] who observed that QTc showed no correlation with BMI before insulin therapy and the change in QTc was not correlated with the change in BMI from before to after insulin therapy. Visceral obesity has been shown to be a more powerful predictor of obesity-related risk and mortality [47], and waist circumference provides a convenient measure of visceral obesity, which could partly explain the result of our study that waist circumference, not BMI, was associated with QTc interval.

The study has several limitations. First, due to its retrospective cross-sectional design, we could not determine temporal or causal relationships between risk factors and prolongation of QTc interval. Second, compared with population-based studies, our study notoriously has a potential for a selection bias. Patients from only one diabetes center were included in the study. Thus, this limits the strength of the study. Third, in the current study, we only measured the QT interval. We did not analysis other QT parameters such as QT dispersion. QT dispersion also has been shown to have a predictive role in all-cause and cardiovascular mortality in type 2 diabetes. Fourthly, since QTc interval was measured before euglycemia was fully achieved, the relatively high plasma glucose value may influence the QTc interval. Ideally, we should have measured them after euglycemia had been achieved completely. Finally, we do not follow up all the subjects to examine the association between prolonged QTc intervals and prevalence of clinical vascular events.

In conclusion, the current study has shown that the prevalence of prolonged QTc interval among Chinese patients with type 2 diabetes is considerably high (30.1%), and that prolongation of QTc interval is associated with height, waist circumference, diastolic blood pressure, postprandial glucose levels, fasting insulin levels and presence of microalbuminuria. These findings have both epidemiological and clinical relevances. They support that patients with type 2 diabetes and prolonged QTc intervals have excess cardiovascular risk and should be treated to reduce the mortality risk.

## Figures and Tables

**Table 1:** Baseline characteristics of the study population.

Factors	QTc > 0.44 s	QTc ≦ 0.44 s	*P* value
	(*n* = 951)	(*n* = 2205)	
Age (years)^†^	55.4 ± 11.0	54.7 ± 10.7	0.070
Sex (male/female)	457/494	1274/931	<0.000
Height (m)^†^	1.64 ± 0.08	1.65 ± 0.08	<0.001
Body weight (kg)^†^	70.0 ± 12.5	70.1 ± 12.0	0.175
BMI (kg/m^2^)^†^	26.1 ± 3.7	25.9 ± 3.2	0.106
Waist circumference^†^	89.6 ± 9.7	88.4 ± 9.5	0.001
Hip circumference^†^	95.3 ± 7.4	94.5 ± 6.6	0.003
WHR^†^	0.94 ± 0.07	0.93 ± 0.07	0.072
Smoking (%)	33.4	36.4	0.109
Duration of diabetes (years)^‡^	4 (1, 8)	3 (0.5, 7)	0.001
HbA1c (%)^†^	8.1 ± 2.1	7.6 ± 1.8	<0.001
Systolic blood pressure (mmHg)^†^	134.0 ± 20.0	130.0 ± 18.8	<0.001
Diastolic blood pressure (mmHg)^†^	76.8 ± 10.1	74.5 ± 9.5	<0.001
Fasting blood glucose (mmol/L)^†^	8.6 ± 2.9	8.6 ± 3.1	0.778
Postprandial blood glucose (mmol/L)^†^	13.8 ± 5.1	12.7 ± 4.7	<0.001
Fasting plasma insulin (*μ*IU/mL)^‡^	7.61 (4.59, 11.99)	7.30 (4.05, 10.31)	<0.001
Postprandial plasma insulin (*μ*IU/mL)^‡^	25.16 (14.81, 46.4)	25.87 (13.73, 45.64)	0.427
Cholesterol (mmol/L)^†^	5.14 ± 1.11	5.02 ± 1.06	0.005
LDL-C (mmol/L)^†^	3.10 ± 1.82	3.00 ± 0.84	0.037
HDL-C (mmol/L)^†^	1.27 ± 0.29	1.26 ± 0.30	0.826
Triglycerides (mg/L)^‡^	1.71 (1.20, 2.3)	1.54 (1.05, 2.30)	<0.001
Microalbuminuria (%)	28.3	20.4	<0.001
Retinopathy (%)	14.1	13.1	0.436
Neuropathy (%)	36.7	36.7	0.984

BMI: body weight index, WHR: waist hip ratio, HbA1c: glycated hemoglobin, TC: total cholesterol, LDL-C: low density lipoprotein cholesterol, and HDL-C: high density lipoprotein cholesterol.

^†^Data are mean ± SD.

^‡^Data are median (quartile).

**Table 2:** Multivariate regression analysis for risk factors of prolonged QTc interval.

	Regression coefficient	OR	95% CI	*P* value
Height	−1.858	0.156	0.032~0.748	0.020
Waist circumference	0.025	1.025	1.010~1.040	0.001
Diastolic blood pressure	0.016	1.016	1.007~1.026	0.000
Postprandial blood glucose	0.040	1.040	1.022~1.059	0.000
Fasting plasma insulin	0.014	1.014	1.003~1.025	0.010
Microalbuminuria	0.236	1.266	1.033~1.551	0.023

## References

[1] Vinik AI, Maser RE, Mitchell BD, Freeman R (2003). Diabetic autonomic neuropathy. *Diabetes Care*.

[2] Maser RE, Mitchell BD, Vinik AI, Freeman R (2003). The association between cardiovascular autonomic neuropathy and mortality in individuals with diabetes a meta-analysis. *Diabetes Care*.

[3] Boulton AJM, Vinik AI, Arezzo JC (2005). Diabetic neuropathies: a statement by the American Diabetes Association. *Diabetes Care*.

[4] Vinik AI, Ziegler D (2007). Diabetic cardiovascular autonomic neuropathy. *Circulation*.

[5] Veglio M, Borra M, Stevens LK, Fuller JH, Perin PC (1999). The relation between QTc interval prolongation and diabetic complications. The EURODIAB IDDM Complication Study Group. *Diabetologia*.

[6] Whitsel EA, Boyko EJ, Siscovick DS (2000). Reassessing the role of QT(c) in the diagnosis of autonomic failure among patients with diabetes: a meta-analysis. *Diabetes Care*.

[7] Tentolouris N, Katsilambros N, Papazachos G (1997). Corrected QT interval in relation to the severity of diabetic autonomic neuropathy. *European Journal of Clinical Investigation*.

[8] Schouten EG, Dekker JM, Meppelink P, Kok FJ, Vandenbroucke JP, Pool J (1991). QT interval prolongation predicts cardiovascular mortality in an apparently healthy population. *Circulation*.

[9] Okin PM, Devereux RB, Lee ET, Galloway JM, Howard BV (2004). Electrocardiographic repolarization complexity and abnormality predict all-cause and cardiovascular mortality in diabetes: the strong heart study. *Diabetes*.

[10] Salles GF, Bloch KV, Cardoso CRL (2004). Mortality and predictors of mortality in a cohort of Brazilian type 2 diabetic patients. *Diabetes Care*.

[11] Rossing P, Breum L, Major-Pedersen A (2001). Prolonged QTc interval predicts mortality in patients with Type 1 diabetes mellitus. *Diabetic Medicine*.

[12] Veglio M, Sivieri R, Chinaglia A, Scaglione L, Cavallo-Perin P (2000). QT interval prolongation and mortality in type 1 diabetic patients: a 5-year cohort prospective study. *Diabetes Care*.

[13] Veglio M, Bruno G, Borra M (2002). Prevalence of increased QT interval duration and dispersion in type 2 diabetic patients and its relationship with coronary heart disease: a population-based cohort. *Journal of Internal Medicine*.

[14] World Health Organization Definition, diagnosis and classification of diabetes mellitus and its complications. Report of a WHO Consultation. Part1: Diagnosis and classification of diabetes mellitus.

[15] Bazett HD (1920). An analysis of the time relations of electrocardiograms. *Heart*.

[16] Fiorentini A, Perciaccante A, Valente R, Paris A, Serra P, Tubani L (2010). The correlation among QTc interval, hyperglycaemia and the impaired autonomic activity. *Autonomic Neuroscience*.

[17] Veglio M, Giunti S, Stevens LK, Fuller JH, Perin PC (2002). Prevalence of Q-T interval dispersion in type 1 diabetes and its relation with cardiac ischemia: the EURODIAB IDDM complications study group. *Diabetes Care*.

[18] Sohaib SMA, Papacosta O, Morris RW, Macfarlane PW, Whincup PH (2008). Length of the QT interval: determinants and prognostic implications in a population-based prospective study of older men. *Journal of Electrocardiology*.

[19] Subbalakshmi NK, Adhikari PM, Sathyanarayana Rao KN, Jeganathan PS (2010). Influencing factors of QTc among the clinical characteristics in type 2 diabetes mellitus. *Diabetes Research and Clinical Practice*.

[20] Festa A, D’Agostino R, Rautaharju P, Mykkänen L, Haffner SM (2000). Relation of systemic blood pressure, left ventricular mass, insulin sensitivity, and coronary artery disease to QT interval duration in nondiabetic and type 2 diabetic subjects. *American Journal of Cardiology*.

[21] Dekker JM, Feskens EJM, Schouten EG, Klootwijk P, Pool J, Kromhout D (1996). QTc duration is associated with levels of insulin and glucose tolerance: the Zutphen elderly study. *Diabetes*.

[22] Ko GTC, Chan JCN, Critchley JAJH, Cockram CS (2000). Cardiovascular disease in Chinese type 2 diabetic women is associated with a prolonged QTc interval. *International Journal of Cardiology*.

[23] Festa A, D’Agostino R, Rautaharju P (1999). Is QT interval a marker of subclinical atherosclerosis in nondiabetic subjects? The Insulin Resistance Atherosclerosis Study (IRAS). *Stroke*.

[24] Kazumi T, Kawaguchi A, Katoh JI, Ikeda Y, Kishi K, Yoshino G (1999). Fasting serum insulin concentrations are associated with QTc duration independent of serum leptin, percent body fat, and BMI. *Diabetes Care*.

[25] Santini V, Ciampittiello G, Gigli F (2007). QTc and autonomic neuropathy in diabetes: effects of acute hyperglycaemia and n-3 PUFA. *Nutrition, Metabolism and Cardiovascular Diseases*.

[26] Passino C, Magagna A, Conforti F (2003). Ventricular repolarization is prolonged in nondipper hypertensive patients: role of left ventricular hypertrophy and autonomic dysfunction. *Journal of Hypertension*.

[27] Grassi G, Esler M (1999). How to assess sympathetic activity in humans. *Journal of Hypertension*.

[28] Xiao HB, Brecker SJ, Gibson DG (1994). Relative effects of left ventricular mass and conduction disturbance on activation in patients with pathological left ventricular hypertrophy. *British Heart Journal*.

[29] Kleiman RB, Houser SR (1989). Outward currents in normal and hypertrophied feline ventricular myocytes. *American Journal of Physiology—Heart and Circulatory Physiology*.

[30] Annila P, Yli-Hankala A, Lindgren L (1993). Effect of atropine on the QT interval and T-wave amplitude in healthy volunteers. *British Journal of Anaesthesia*.

[31] Marfella R, Nappo F, de Angelis L, Paolisso G, Tagliamonte MR, Giugliano D (2000). Hemodynamic effects of acute hyperglycemia in type 2 diabetic patients. *Diabetes Care*.

[32] Tesfamariam B, Cohen RA (1992). Free radicals mediate endothelial cell dysfunction caused by elevated glucose. *American Journal of Physiology—Heart and Circulatory Physiology*.

[33] Giugliano D, Ceriello A, Paolisso G (1996). Oxidative stress and diabetic vascular complications. *Diabetes Care*.

[34] Marfella R, Nappo F, de Angelis L, Siniscalchi M, Rossi F, Giugliano D (2000). The effect of acute hyperglycaemia on QTc duration in healthy man. *Diabetologia*.

[35] Marfella R, Verrazzo G, Acampora R (1995). Glutathione reverses systemic hemodynamic changes induced by acute hyperglycemia in healthy subjects. *American Journal of Physiology—Endocrinology and Metabolism*.

[36] Takebayashi K, Naruse R, Morita K, Aso Y, Inukai T (2012). The effect of insulin therapy and plasma glucose levels on corrected QT intervals in patients with type 2 diabetes. *Journal of Clinical Medicine and Research*.

[37] Gerstein HC, Miller ME, Byington RP (2008). Effects of intensive glucose lowering in type 2 diabetes. *The New England Journal of Medicine*.

[38] Gastaldelli A, Emdin M, Conforti F, Camastra S, Ferrannini E (2000). Insulin prolongs the QTc interval in humans. *American Journal of Physiology—Regulatory Integrative and Comparative Physiology*.

[39] van Noord C, Sturkenboom MCJM, Straus SMJM (2010). Serum glucose and insulin are associated with QTc and RR intervals in nondiabetic elderly. *European Journal of Endocrinology*.

[40] Earle KA, Mishra B, Morocutti A, Barnes D, Chambers J, Viberti GC (2000). QT dispersion in microalbuminuric Type 1 diabetic patients without myocardial ischemia. *Journal of Diabetes and its Complications*.

[41] Meier M, Muhr D, Weiss M, Tatsch K, Standl E, Schnell O (2000). QTc interval and scintigraphically assessed myocardial perfusion in newly diagnosed and long-term type 1 diabetes mellitus. *Journal of Diabetes and its Complications*.

[42] Christensen PK, Gall MA, Major-Pedersen A (2000). QTc interval length and QT dispersion as predictors of mortality in patients with non-insulin-dependent diabetes. *Scandinavian Journal of Clinical and Laboratory Investigation*.

[43] Rutter MK, Viswanath S, McComb JM, Kesteven P, Marshall SM (2002). QT prolongation in patients with Type 2 diabetes and microalbuminuria. *Clinical Autonomic Research*.

[44] Jensen T, Borch-Johnsen K, Kofoed-Enevoldsen A, Deckert T (1987). Coronary heart disease in young Type 1 (insulin-dependent) diabetic patients with and without diabetic nephropathy: incidence and risk factors. *Diabetologia*.

[45] Witte DR, Tesfaye S, Chaturvedi N, Eaton SEM, Kempler P, Fuller JH (2005). Risk factors for cardiac autonomic neuropathy in type 1 diabetes mellitus. *Diabetologia*.

[46] El-Gamal A, Gallagher D, Nawras A (1995). Effects of obesity on QT, RR, and QTc intervals. *American Journal of Cardiology*.

[47] Montague CT, O’Rahilly S (2000). The perils of portliness: causes and consequences of visceral adiposity. *Diabetes*.

